# Targeted deletion of Pf prophages from diverse *Pseudomonas aeruginosa* isolates has differential impacts on quorum sensing and virulence traits

**DOI:** 10.1128/jb.00402-23

**Published:** 2024-04-30

**Authors:** Amelia K. Schmidt, Caleb M. Schwartzkopf, Julie D. Pourtois, Elizabeth B. Burgener, Dominick R. Faith, Alex Joyce, Tyrza Lamma, Geetha Kumar, Paul L. Bollyky, Patrick R. Secor

**Affiliations:** 1 Division of Biological Sciences, University of Montana, Missoula, Montana, USA; 2 Department of Medicine, Division of Infectious Diseases and Geographic Medicine, Stanford University School of Medicine, Stanford, California, USA; 3 Division of Pediatric Pulmonology and Sleep Medicine, Children’s Hospital of Los Angeles, Keck School of Medicine, University of Southern California, Los Angeles, California, USA; 4 Center for Excellence in Pulmonary Biology, Department of Pediatrics, Stanford University, Stanford, California, USA; 5 School of Biotechnology, Amrita Vishwa Vidyapeetham, Amritapuri, Kerala, India; University of California San Francisco, San Francisco, California, USA

**Keywords:** *Pseudomonas aeruginosa*, Filamentous Pf bacteriophage, quorum sensing, biofilms, pyocyanin, *C. elegans*, prophage, virulence

## Abstract

*Pseudomonas aeruginosa* is an opportunistic bacterial pathogen that commonly causes medical hardware, wound, and respiratory infections. Temperate filamentous Pf phages that infect *P. aeruginosa* impact numerous virulence phenotypes. Most work on Pf phages has focused on Pf4 and its host *P. aeruginosa* PAO1. Expanding from Pf4 and PAO1, this study explores diverse Pf phages infecting *P. aeruginosa* clinical isolates. We describe a simple technique targeting the Pf lysogeny maintenance gene, *pflM* (*PA0718*), that enables the effective elimination of Pf prophages from diverse *P. aeruginosa* hosts. The *pflM* gene shows diversity among different Pf phage isolates; however, all examined *pflM* alleles encode the DUF5447 domain. We demonstrate that *pflM* deletion results in prophage excision but not replication, leading to total prophage loss, indicating a role for lysis/lysogeny decisions for the DUF5447 domain. This study also assesses the effects different Pf phages have on host quorum sensing, biofilm formation, pigment production, and virulence against the bacterivorous nematode *Caenorhabditis elegans*. We find that Pf phages have strain-specific impacts on quorum sensing and biofilm formation, but nearly all suppress pigment production and increase *C. elegans* avoidance behavior. Collectively, this research not only introduces a valuable tool for Pf prophage elimination from diverse *P. aeruginosa* isolates but also advances our understanding of the complex relationship between *P. aeruginosa* and filamentous Pf phages.

**IMPORTANCE:**

*Pseudomonas aeruginosa* is an opportunistic bacterial pathogen that is frequently infected by filamentous Pf phages (viruses) that integrate into its chromosome, affecting behavior. Although prior work has focused on Pf4 and PAO1, this study investigates diverse Pf in clinical isolates. A simple method targeting the deletion of the Pf lysogeny maintenance gene *pflM* (*PA0718*) effectively eliminates Pf prophages from clinical isolates. The research evaluates the impact Pf prophages have on bacterial quorum sensing, biofilm formation, and virulence phenotypes. This work introduces a valuable tool to eliminate Pf prophages from clinical isolates and advances our understanding of *P. aeruginosa* and filamentous Pf phage interactions.

## INTRODUCTION

*Pseudomonas aeruginosa* is an opportunistic bacterial pathogen that commonly infects medical hardware, diabetic ulcers, burn wounds, and the airways of cystic fibrosis patients (1). *P. aeruginosa* isolates are often infected by filamentous viruses (phages) called Pf (2–4). Pf phages live a temperate lifestyle and integrate into the bacterial chromosome as a prophage, passively replicating with each bacterial cell division. When induced, the Pf prophage is excised from the chromosome, forming a circular double-stranded episome called the replicative form (5). Pf replicative form copy numbers increase in the cytoplasm where they serve as templates for viral transcription. The replicative form is also a template for rolling circle replication, which generates circular single-strand DNA genomes that are packaged into filamentous virions as they are extruded from the cell (3, 6).

Filamentous Pf virions are associated with enhanced *P. aeruginosa* virulence potential by promoting biofilm formation (7) and inhibiting phagocytic uptake by macrophages (8, 9). Pf virions also carry a high negative charge density allowing them to sequester cationic antimicrobials such as aminoglycoside antibiotics and antimicrobial peptides (7, 10, 11). Additionally, Pf phages enhance the virulence potential of *P. aeruginosa* by modulating the secretion of the quorum-regulated virulence factor pyocyanin (12, 13). These properties may explain why the presence of Pf virions at sites of infection is associated with more chronic lung infections and antibiotic resistance in cystic fibrosis patients (8) and why *P. aeruginosa* strains cured of their Pf infection are less virulent in murine models of pneumonia (14) and wound infection (15).

Most studies to date have focused on interactions between Pf isolate Pf4 and its host *P. aeruginosa* PAO1 (3, 9, 11, 14). Despite the clear link between Pf4 and the virulence of *P. aeruginosa* PAO1, the effects of diverse Pf that infect *P. aeruginosa* clinical isolates have on virulence phenotypes remain unclear. This is in part due to the significant challenge of “curing” clinical isolates of their Pf prophage infections. Prior efforts to delete Pf4 from PAO1 relied on the integration of a selectable marker into the integration site used by Pf4 (14), which precludes complementation studies that re-introduce the Pf4 prophage to the host chromosome. In prior work, we were able to generate a clean Pf4 deletion strain by first deleting the *pfiTA* toxin-antitoxin module encoded by Pf4, followed by deletion of the rest of the prophage (16).

Here, we find that the Pf4 gene *PA0718* maintains Pf4 in a lysogenic state; we therefore refer to *PA0718* as the Pf lysogeny maintenance gene *pflM*. Deletion of *PA0718* or homologous alleles from Pf prophages in clinical *P. aeruginosa* isolates, LESB58, CPA0053, and CPA0087, and the multidrug-resistant strain DDRC3 resulted in the complete loss of Pf prophages from each strain. Furthermore, we observed that some substrains of PAO1 are lysogenized by two Pf phages, Pf4 and Pf6, and we successfully cured PAO1 of both Pf4 and Pf6 prophages. We compare phenotypic differences between wild-type and ∆Pf prophage mutants by assessing Las, Rhl, and PQS quorum-sensing activity, biofilm formation, and pyocyanin production. We also examine how Pf prophages impact virulence phenotypes in a *C. elegans* avoidance model. Overall, we present a new methodology for efficiently curing *P. aeruginosa* strains of their resident Pf prophages and leverage this tool to gain insight into the diverse impacts Pf phages have on their bacterial hosts.

## RESULTS

### PA0718 (PflM) maintains Pf4 lysogeny in *P. aeruginosa* PAO1

**Fig 1:**
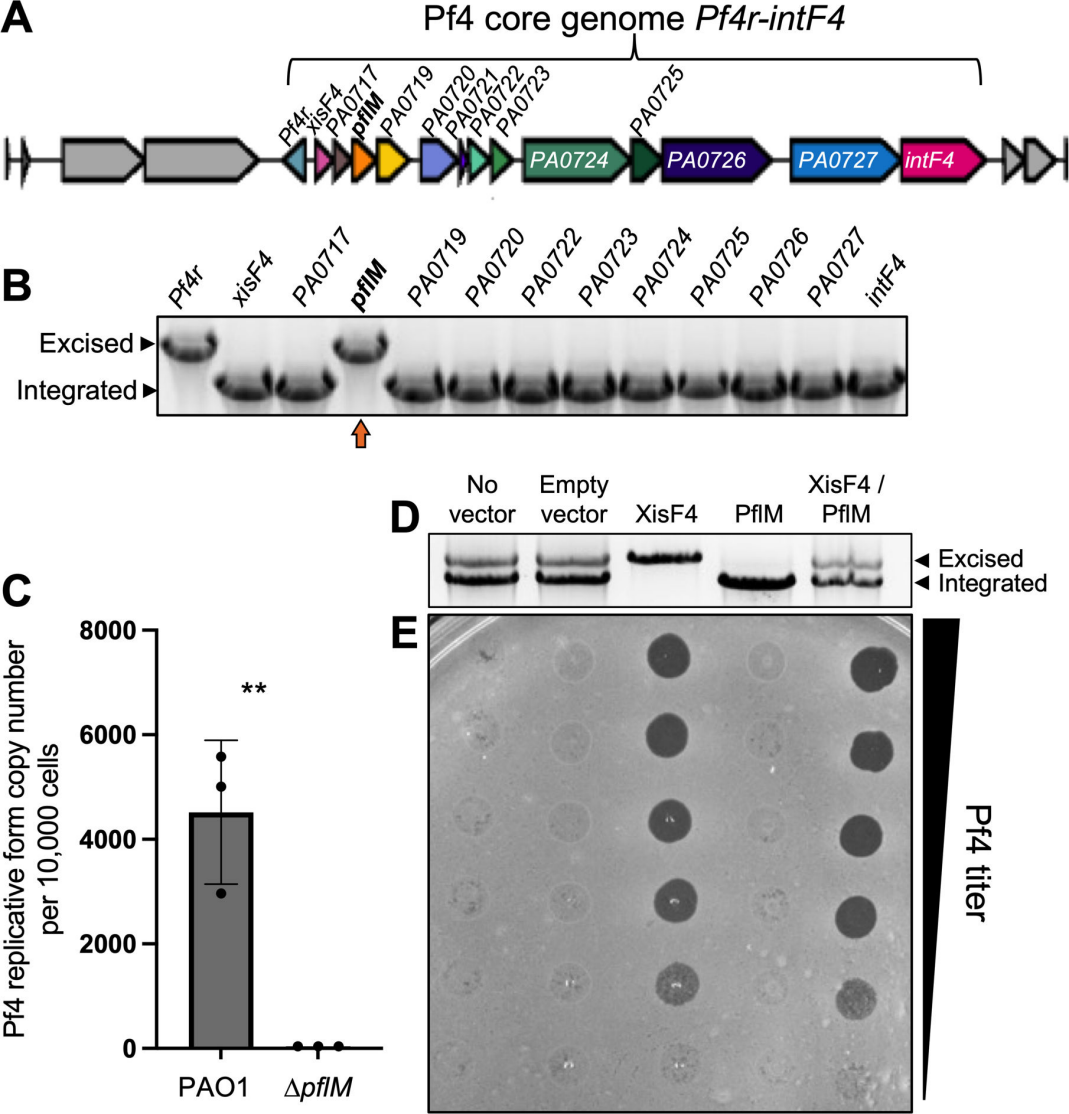
The Pf4 phage gene *PA0718* (*pflM*) maintains lysogeny. (**A**) The Pf4 prophage is shown. (**B**) Multiplex PCR was used to measure Pf4 prophage integration and excision from the PAO1 chromosome in Pf4 single-gene mutants, which were generated through allelic exchange. Deletion of the Pf4 repressor (*Pf4r*) and *PA0718* results in prophage excision. Despite our best efforts, we were unable to generate a single-gene mutant of Pf4 gene *pfsE,* marked with an asterisk. (**C**) Quantitative PCR (qPCR) was used to measure episomal Pf4 replicative form in cells after 18 hours of growth in lysogeny broth (LB) broth. Data are the mean ± SEM of three replicate experiments. The lower limit of detection for the assay is 37.85 copy numbers per 10,000 cells. **P < 0.01, Student’s *t*-test. (**D**) PA0718 and/or XisF4 were expressed from an inducible plasmid in *P. aeruginosa* PAO1. After 18 hours, Pf4 integration and excision were measured by excision assay. (**E**) Pf4 virions in filtered supernatants collected from the indicated strains were titered on lawns of *P. aeruginosa* ∆Pf4. A representative image is shown.

While performing single-gene deletions from the core Pf4 genome (*pf4r-intF4*) in *P. aeruginosa* PAO1 (Fig. 1A), we noted that deleting either the *Pf4r* repressor or the *PA0718* gene results in the complete excision of the Pf4 prophage from the *P. aeruginosa* chromosome (Fig. 1B, upper bands). Prior work demonstrates that deletion of the *Pf4r* repressor induces Pf4 prophage excision and virion replication (17), but how *PA0718* is involved in Pf4 excision is not known.

After excision, Pf4 replicates as a circular episome called the replicative form (5). We used qPCR to measure circular Pf4 replicative form copy numbers in wild-type and ∆*PA0718* cells. In wild-type cells, approximately 4,400 replicative form copies were detected for every 10,000 cells; however, the Pf4 replicative form was not detected in ∆*PA0718* cells (Fig. 1C), indicating that Pf4 genome replication is not initiated, and the replicative form is lost as cells divide. These results indicate that PA0718 maintains the Pf4 prophage in a lysogenic state and that deleting *PA0718* induces Pf4 prophage excision, but not replication, curing PAO1 of its Pf4 infection. Herein, we refer to *PA0718* as the Pf lysogeny maintenance gene *pflM*.

The observation that 4,400 Pf4 replicative form copies are detected for every 10,000 wild-type cells (Fig. 1C) indicates that Pf4 is actively replicating in a subpopulation of cells. We used a multiplex PCR excision assay to measure Pf4 prophage excision and integration in *P. aeruginosa* populations. In PAO1 populations with no expression vector or those carrying an empty expression vector, both Pf4 prophage integration and excision are observed (Fig. 1D, two bands are present); however, infectious virions were not detected in supernatants by plaque assay, suggesting that Pf4 is replicating at low levels during planktonic growth in LB broth, consistent with prior results (18).

The Pf4 excisionase XisF4 regulates Pf4 prophage excision (17), and expressing XisF4 in *trans* induces complete Pf4 prophage excision (Fig. 1D) and robust virion replication (Fig. 1E). In contrast, expressing PflM in *trans* maintains the entire population in a lysogenic state (Fig. 1D), and virion replication is not detected (Fig. 1E). When PflM and XisF4 are expressed together, both Pf4 integration and excision products are observed (Fig. 1D), and infectious virions are produced at titers comparable with cells where XisF4 was expressed by itself (Fig. 1E). These results indicate that expressing PflM is not sufficient to inhibit XisF4-mediated Pf4 prophage excision and replication but that PflM can maintain some cells in a lysogenic state during active viral replication.

### The targeted deletion of *pflM* cures diverse *P. aeruginosa* isolates of their Pf prophages

We hypothesized that deleting *pflM* would provide a convenient way to cure *P. aeruginosa* clinical isolates of their Pf prophages. To test this hypothesis, we deleted *pflM* from the Pf prophages in cystic fibrosis isolate LESB58, two cystic fibrosis isolates from the Stanford Cystic Fibrosis Clinic (CPA0053 and CPA0087), and the multidrug-resistant urine isolate DDRC3 (Table 1).

**TABLE 1:** *P. aeruginosa* isolates and Pf prophage characteristics

Strain	Accession	Source	Mucoid?	Pf name/lineage	Pf integration site	Pf prophage length (kb)
PAO1	GCF_000006765.1	Lab strain	No	Pf4/I	tRNA-Gly	12.4
				Pf6/I	tRNA-Met	12.1
LESB58	FM209186.1	CF isolate, Liverpool, United Kingdom	No	Pf-LESB58/II	Direct repeat	10.5
CPA0053	CP137561	CF isolate, Stanford, CA, USA	No	Pf-CPA0053/II	Direct repeat	10.4
CPA0087	CP137562	CF isolate, Stanford, CA, USA	No	Pf-CPA0087/II	tRNA-Gly	11.1
DDRC3	CP137563	Urine isolate, Trivandrum, Kerala, India	No	Pf-DDRC3/II	tRNA-Gly	15.5

**Fig 2:**
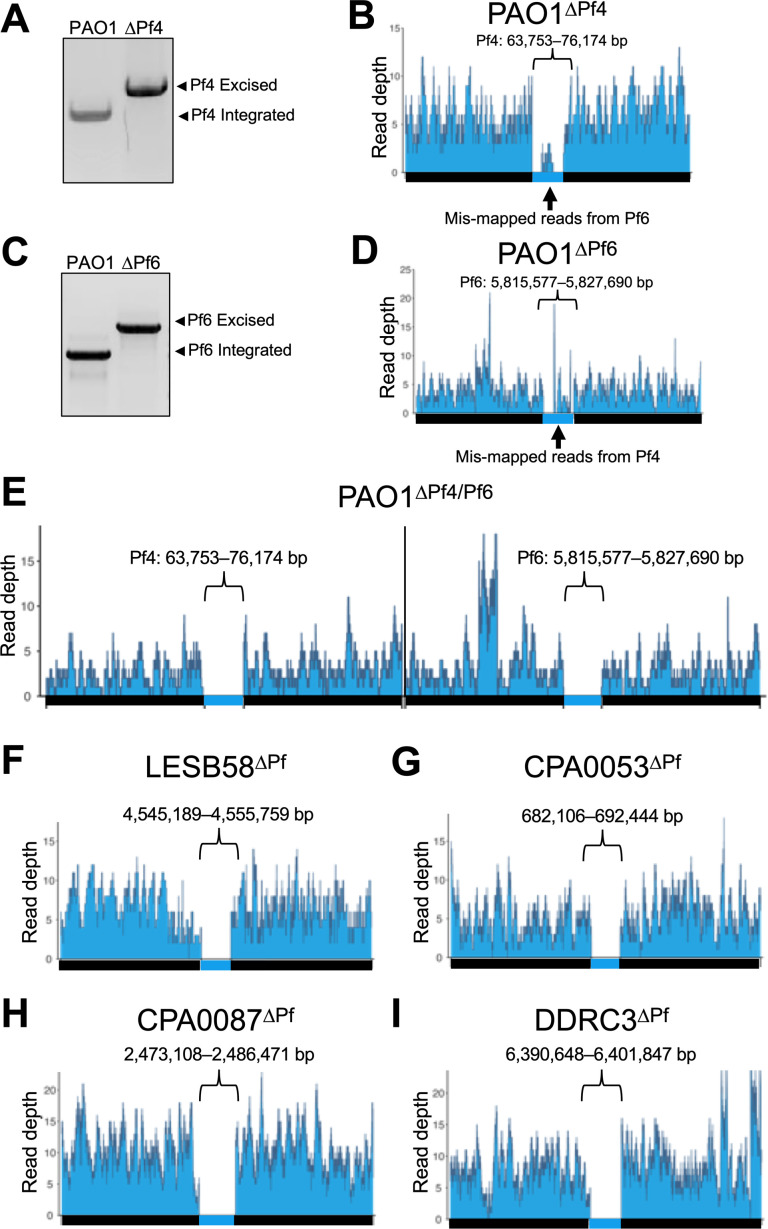
Targeted deletion of *pflM* cures diverse *P. aeruginosa* isolates of their Pf prophage infections. A multiplex PCR assay and long-read whole genome sequencing were used to confirm the loss of (**A and B**) the Pf4 prophage or (**C and D**) the Pf6 prophage from the PAO1 chromosome. (**E-I**) Long-read whole genome sequencing was used to confirm the successful deletion of the indicated Pf prophages. Reads were aligned to 50 kb sequences flanking the Pf prophage insertion sites in the parental chromosome. The genomic coordinates for each Pf prophage are shown above each bracket.

Pf prophage loss in PAO1 was screened by multiplex PCR (Fig. 2A/C) and confirmed in all strains by long-read whole-genome sequencing. Targeting *pflM* successfully cured all the above clinical *P. aeruginosa* isolates of their Pf prophages (Fig. 2B, D through G). Of the Pf prophages we deleted, four were integrated into tRNA genes (three in tRNA-Gly and one in tRNA-Met), and two were integrated into direct repeats (Table 1). Furthermore, Pf prophages fall into two main lineages (I and II, Table 1) (4), and we were successful in deleting representatives from each lineage with an efficiency rate of approximately 17%. These observations indicate neither integration site nor lineage has an influence on *pflM*-mediated Pf prophage deletion.

To determine if prophage loss impacts bacterial growth, we performed growth curves over 18 hours and compared parental and prophage mutant strains (Fig. S1). In strains PAO1, CPA0053, and DDRC3, deletion of the Pf prophage has no significant impact on bacterial growth (Fig. S1A through C). For strains CPA0087 and LESB58, prophage presence significantly reduces or increases growth rates, respectively (*P* < 0.0001) (Fig. S1D through E). The decreased growth of strain CPA0087^∆Pf^ may be related to the large increase in pyocyanin production, which could negatively impact bacterial growth. The increased growth rate of LESB58^∆Pf^ may be explained by relieving the metabolic burden of Pf phage replication. Alternatively, differences in Pf prophage insertion loci in different *P. aeruginosa* strains could affect bacterial growth dynamics.

Many *P. aeruginosa* strains are infected by one or more Pf prophages (3). For example, some *P. aeruginosa* PAO1 sub-isolates are infected by Pf4 and Pf6 (19). Deleting *pflM* from Pf4 results in the loss of the Pf4 prophage, as does deleting *pflM* from Pf6 (Fig. 2B and D). Furthermore, we were able to delete Pf6 from ∆Pf4, producing a PAO1^∆Pf4/Pf6^ double mutant (Fig. 2E). This observation indicates that *pflM* from one Pf prophage is specific to that prophage and does not compensate for the loss of *pflM* from another Pf prophage residing in the same host.

**Fig 3:**
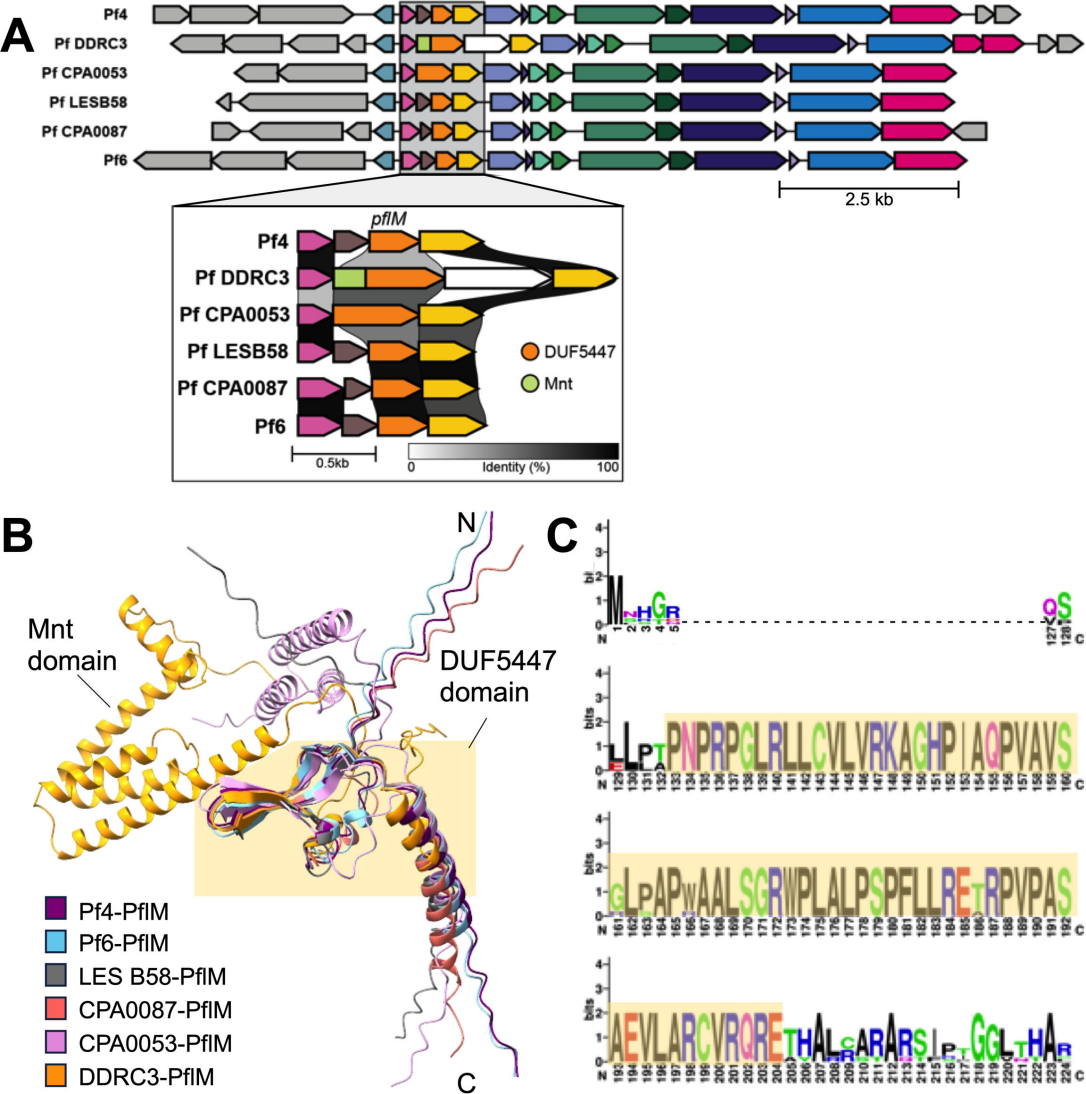
Sequence and structural analysis of PflM. (**A**) Pf prophage annotation was performed with Rapid Annotation using Subsystem Technology (RAST). Inset: Clinker was used to generate alignments of the *pflM* loci spanning from *xisF* to *PA0719* in the Pf4 reference sequence. DUF5447 and mnt domains in *pflM* are indicated by orange or green, respectively, in the inset. (**B**) PflM structures were predicted using AlphaFold2and aligned using ChimeraX. The highlighted region indicates the DUF5447 domain and the Mnt domain is indicated for the PflM sequence from strain DDRC3. (**C**) A protein sequence logo for PflM was generated with WebLogo3 (v. 2.8.2); dashes represent variable residues. The DUF5447 domain is highlighted.

PflM specificity may be explained by the diversity in the operon encoding *pflM* or in the *pflM* allele itself (Fig. 3A). All *pflM* sequences examined contain a predicted DUF5447 domain (pfam17525), which, as pointed out in prior work, appears to originate from a disrupted gene encoding the Mnt (PHA01513) domain (20). The Mnt protein encoded by *Salmonella* phage P22 governs lysis-lysogeny decisions by binding phage operator sequences (21, 22), suggesting that PflM may regulate Pf lysis-lysogeny decisions by a similar mechanism. In the Pf DDRC3 *pflM* allele, the Mnt domain is present and is fused to the DUF5447 domain, whereas in Pf4, Pf LESB58, Pf CPA0087, and Pf6, *pflM* is truncated by a 5’ insertion of a gene of unknown function (*PA0717* in Pf4) (Fig. 3A). Structural prediction and sequence alignments indicate that despite the different isoforms PflM present within different Pf prophages, residues that are predicted to form an anti-parallel β-sheet characteristic of the DUF5447 domain are conserved (Fig. 3B and C).

### Pf phages differentially modulate host quorum sensing

Pf4 is known to suppress PQS and Rhl quorum sensing in *P. aeruginosa* PAO1 (12, 13, 23). We hypothesized that Pf phages would likewise modulate quorum sensing in the Pf deletion strains we constructed here. To test this, we used fluorescent transcriptional reporters (12) to measure Las (*rsaL*), Rhl (*rhlA*), and PQS (*pqsA*) transcriptional activity in parental strains and Pf mutants.

**Fig 4:**
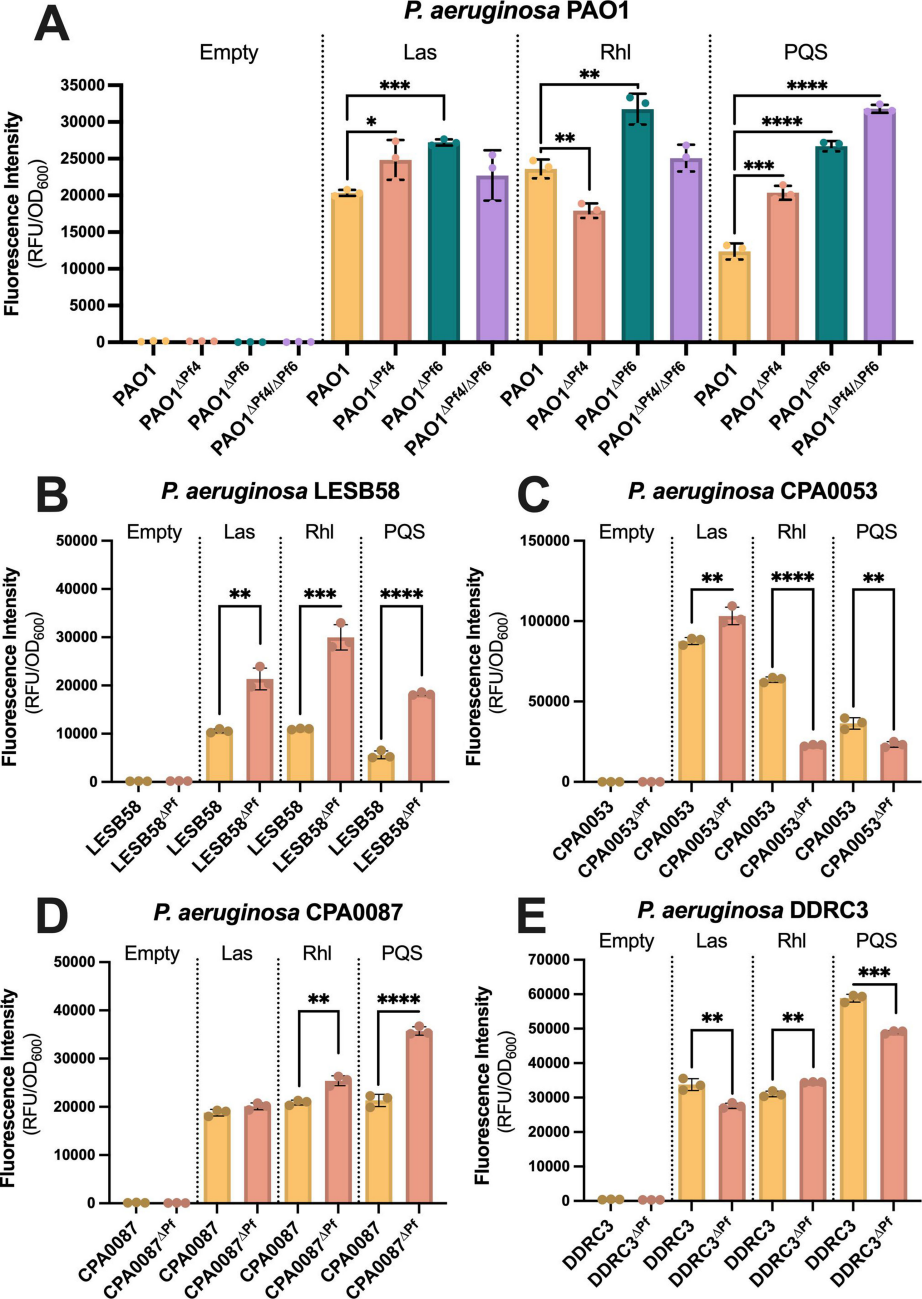
Pf phage differentially modulates *P. aeruginosa* quorum sensing. Fluorescence from the transcriptional reporters P*_rsaLI_-gfp* (Las), P*_rhlA_-gfp* (Rhl), or P*_pqsA_-gfp* (PQS) was measured in the indicated strains after 18 hours of growth. Reporter fluorescence intensity was normalized to cell density (OD_600_) at each time point. Data are the mean ± SEM of seven replicates. *P < 0.05, **P < 0.01, ***P < 0.001, ****P < 0.0001, Student’s *t*-test comparing ∆Pf strains with the wild-type parent at each time point.

Over an 18-hour growth period, we find that differences in Las, Rhl, and PQS quorum-sensing activity vary by Pf phage and *P. aeruginosa* host (Fig. S8). The general trends for each quorum-sensing reporter hold over 18 hours; however, for clarity, we show only the 18-hour time point in Fig. 4. After 18 hours, PAO1^∆Pf4^ Las and PQS signaling are significantly upregulated (*P* < 0.01 and *P* < 0.0001, respectively), whereas Rhl transcription is downregulated (Fig. 4A). Pf6 differentially affects PAO1 quorum sensing—Las, Rhl, and PQS signaling are all upregulated in PAO1^∆Pf6^ compared with the parental strain (Fig. 4A). Deleting both Pf4 and Pf6 had no significant impact on Las or Rhl signaling, but PQS signaling was significantly upregulated in PAO1^∆Pf4/Pf6^ compared with the parental strain (Fig. 4A), which is consistent with prior work (12, 24).

After 18 hours, PQS transcriptional activity is also significantly (*P* < 0.001) upregulated in LESB58^∆Pf^, as are Las and Rhl (Fig. 4B). In strain CPA0053^∆Pf^, Las signaling was significantly upregulated (*P* < 0.001), whereas Rhl and PQS transcription is reduced when the Pf prophage is deleted (Fig. 4C). PQS and Rhl are upregulated in CPA0087^∆Pf^, whereas Las and signaling are not significantly affected (Fig. 4D). Finally, in DDRC3^∆Pf^, Las and PQS signaling are significantly downregulated (*P* < 0.001), whereas Rhl signaling is significantly upregulated (*P* < 0.001) (Fig. 4E). Taken together, these data indicate that Pf phages have diverse and complex relationships with host quorum-sensing pathways that vary significantly by strain.

### Pf phages have contrasting impacts on *P. aeruginosa* biofilm formation

**Fig 5:**
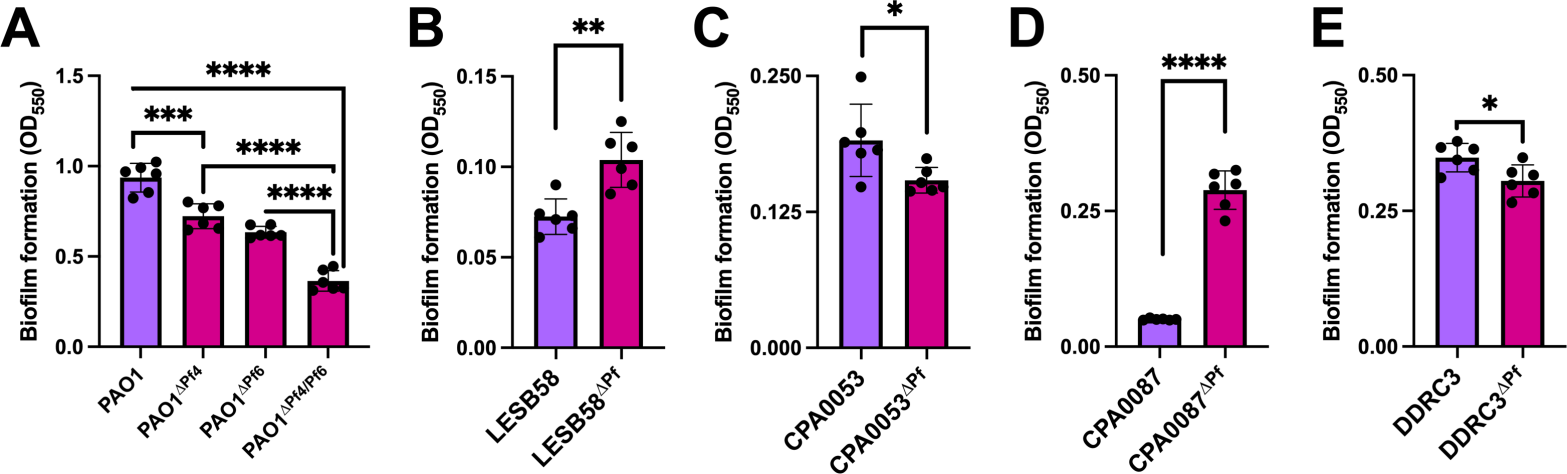
Pf prophage deletion has significant but variable effects on *P. aeruginosa* biofilm formation. Crystal violet biofilm assays were performed to measure biofilm formation of the indicated strains after 48 hours of incubation. Data are the mean ± SEM of six replicates. *P < 0.05, **P < 0.01, ***P < 0.001, ****P < 0.0001, Student’s *t*-test.

Pf4 is known to promote *P. aeruginosa* PAO1 biofilm assembly and function (3, 5, 7, 11, 14, 25, 26). To test if other Pf isolates similarly affect biofilm formation, we used the crystal violet biofilm assay (27) to measure the biofilm formation of lysogenized *P. aeruginosa* isolates compared with the Pf prophage deletion mutants. In PAO1, deletion of either Pf4 or Pf6 significantly (*P* < 0.001) reduced biofilm formation by 1.79-fold and 2.33-fold, respectively, whereas deletion of both Pf4 and Pf6 reduced biofilm formation by 7.14-fold (Fig. 5A). This result indicates both Pf4 and Pf6 contribute to PAO1 biofilm formation, which is consistent with prior observations (5, 7, 11, 14, 25, 26). The clinical isolates in general did not form as robust biofilms as the PAO1 laboratory strain under the *in vitro* conditions tested. Even so, deleting the Pf prophage from strains CPA0053 and DDRC3 modestly but significantly (*P* < 0.05) reduced biofilm formation (Fig. 5C and E). In contrast, biofilm formation was significantly (*P* < 0.01) increased in strains LESB58^∆Pf^ and CPA0087^∆Pf^ compared with the parental strains (Fig. 5B and D). The variation in biofilm formation phenotypes is perhaps not surprising, given the variation inherent in the strains examined here, ranging from lab-adapted strains to CF-lung-adapted *P. aeruginosa* isolates. Although quorum-sensing regulation varies between Pf lysogens and their corresponding Pf prophage mutants, there is no clear trend of modified QS expression and biofilm formation (Fig. 5).

### Pf phages suppress *P. aeruginosa* pyocyanin production in most clinical isolates

**Fig 6:**
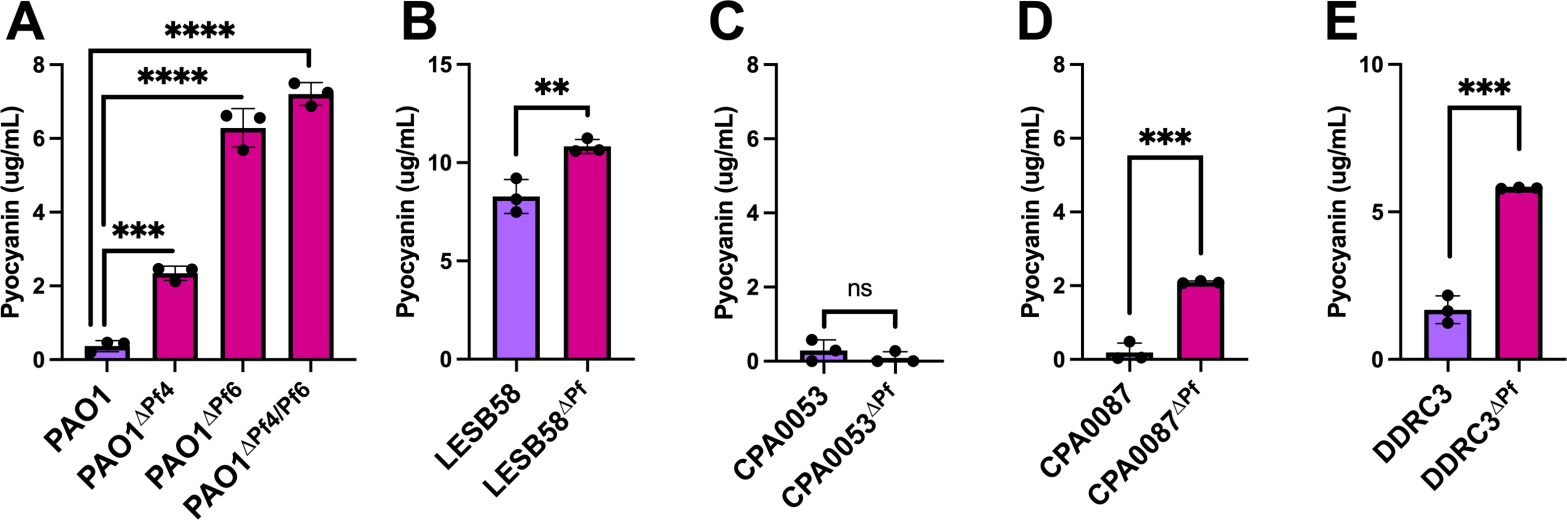
Pyocyanin production is enhanced in Pf prophage deletion strains. (**A-E**) Pyocyanin was CHCl_3_-HCl extracted from the supernatants of the indicated cultures after 18 hours of incubation. Pyocyanin concentration was measured (Abs 520 nm). Data are the mean ± SEM of three replicates. **P < 0.01, ***P < 0.001, ****P < 0.0001, Student’s *t*-test.

Pyocyanin is a redox-active quorum-regulated virulence factor (28). Deleting the Pf4 prophage from PAO1 enhances pyocyanin production (12). We observed increased pyocyanin production in all ∆Pf strains tested except CPA0053, which did not produce much pyocyanin under any condition tested (Fig. 6A through E). We confirmed that neither the *pqsABCD* operon nor *pqsR* encoded by CPA0053 is mutated, nor is *lasI, lasR, rhlI,* or *rhlR*, indicating that other factors/signaling pathways contribute to reduced pyocyanin production by this strain. These results suggest that some Pf prophages encode gene(s) that inhibit host pyocyanin production.

### Pf phages induce avoidance behavior in bacterivorous nematodes

**Fig 7:**
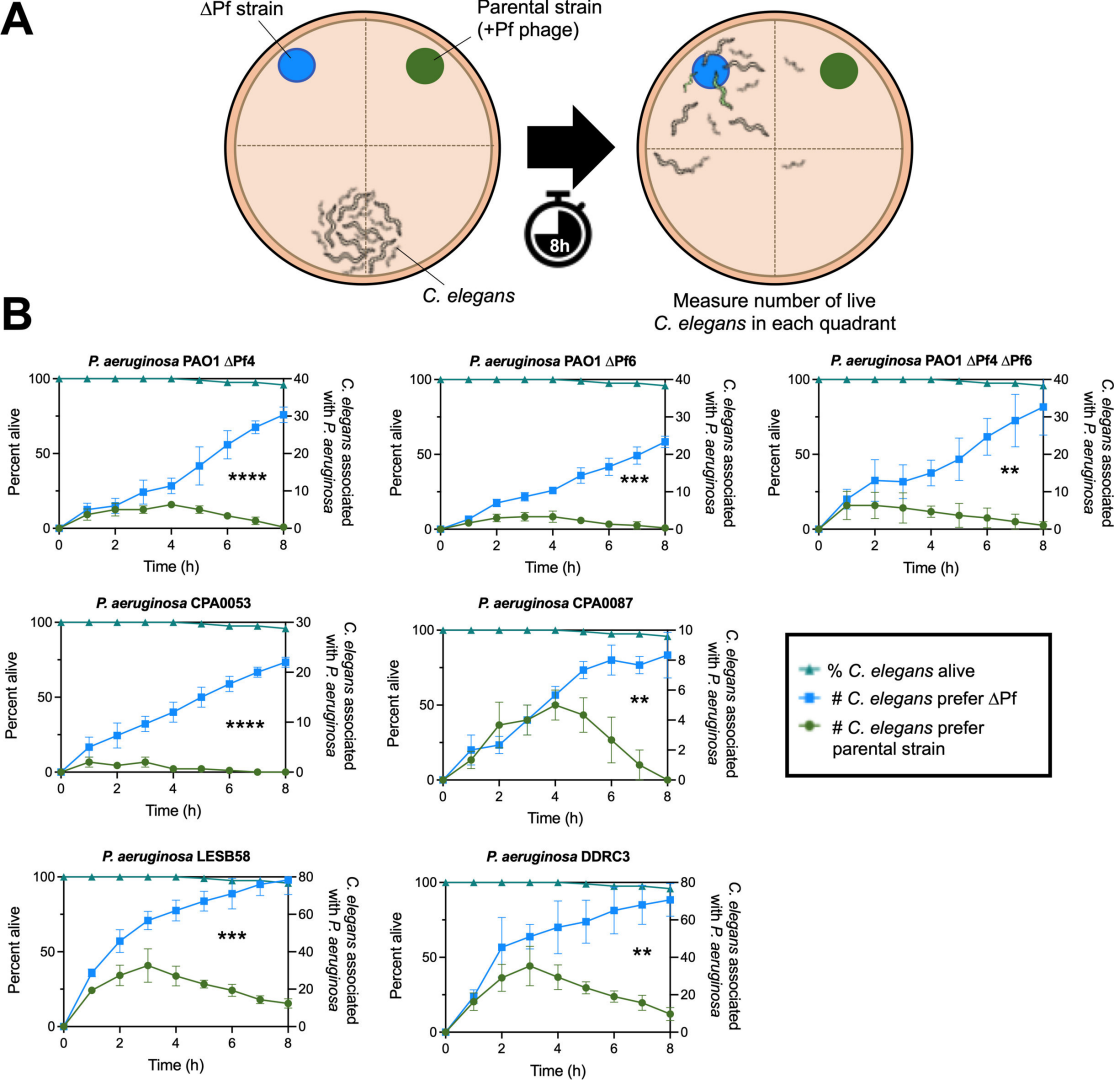
*C. elegans* actively avoids *P. aeruginosa* Pf lysogens. (**A**) Experimental design: *P. aeruginosa* and isogenic ∆Pf mutants were spotted on normal nematode growth medium (NNGM) plates with wild-type N2 *C. elegans* at the indicated locations. *C. elegans* localization to the indicated quadrants was measured hourly. (**B**) *C. elegans* association with *P. aeruginosa* (circles) or isogenic ∆Pf mutants (squares) in the indicated strain backgrounds was measured hourly over 8 hours (three experiments with *N* = 30 per replicate [90 animals total]). *P* values were calculated by two-way ANOVA (analysis of variance) comparing ∆Pf strains with the parental strains using the Šidák correction (95% confidence interval threshold), **P < 0.01, ***P < 0.001, ****P < 0.0001.

In the environment, bacterivores impose high selective pressures on bacteria (29, 30). Pf4 modulation of quorum-regulated virulence factors increases *P. aeruginosa* fitness against the bacterivorous nematode *C. elegans* (12). We hypothesized that Pf prophages in *P. aeruginosa* clinical isolates would similarly protect *P. aeruginosa* from predation by *C. elegans*. To test this, we employed *C. elegans* avoidance assays (31–34) as a metric of bacterial fitness when confronted with nematode predation (Fig. 7A). *C. elegans* avoided all Pf lysogens, preferring to associate with the ∆Pf strains in every case (Fig. 7B). Note that nematode survival was over 95% over the course of the experiment (8 hours) in all experiments (Fig. 7B, triangles). Collectively, our results suggest that Pf modulates *P. aeruginosa* virulence phenotypes in ways that repel *C. elegans*.

## DISCUSSION

This study describes a convenient method to cure *P. aeruginosa* isolates of their Pf prophage infections and explores relationships between diverse Pf phages and their *P. aeruginosa* hosts. Overall, different Pf isolates exhibit varying effects on host quorum sensing and biofilm formation. One commonality between all Pf isolates examined is their ability to suppress pyocyanin production and repel *C. elegans* away from *P. aeruginosa*, protecting their host from predation.

Our results indicate that PflM maintains Pf in a lysogenic state and that deleting the *pflM* gene induces Pf prophage excision, but not replication. In Pf4, the site-specific tyrosine recombinase IntF4 catalyzes Pf4 prophage integration into and excision from the chromosome, whereas the Pf4 excisionase XisF4 regulates Pf4 prophage excision by promoting interactions between IntF4 and Pf4 attachment sites as well as inducing the expression of the replication initiation protein PA0727 (4, 17). In response to stimuli such as oxidative stress (35), these coordinated events induce Pf4 prophage excision and initiate episomal replication, allowing Pf4 to complete its lifecycle.

Although it is presently not known how PflM maintains Pf lysogeny, it is possible that PflM regulates the IntF recombinase or inhibits the XisF excisionase, causing the Pf prophage to excise from the chromosome without concurrently inducing the replication initiation protein PA0727 (6), thus resulting in Pf prophage excision without initiating episomal Pf replication. Additionally, the DUF5447 domain (pfam17525) encoded by PflM is found only in Pseudomonads, indicating that the mechanism of PflM in maintaining Pf lysogeny is unique, compared with other lysogeny maintenance mechanisms employed by other phages.

Our study highlights the role of Pf phages in manipulating *P. aeruginosa* quorum sensing. Pf phages have varying effects on host quorum sensing; broadly, we determine that Pf phage modulates quorum sensing activity and quorum-regulated phenotypes in all bacterial strains tested. These findings imply that different Pf isolates interact with host quorum-sensing networks in diverse ways, indicating a complex interplay between Pf phages and host regulatory systems.

Quorum sensing regulates *P. aeruginosa* biofilm formation and Pf4 contributes to biofilm formation in PAO1 (5, 7, 14, 18, 36). Consistently, we find that both Pf4 and Pf6 contribute to PAO1 biofilm formation. Interestingly, the impact of Pf prophage deletion on biofilm formation varies among clinical isolates, which may be related to different quorum-sensing hierarchies present in clinical P. *aeruginosa* isolates (37). Alternatively, Pf prophage encodes accessory genes that may modulate host quorum-sensing systems.

Despite differences in interactions between Pf phages and host quorum sensing, deleting Pf prophages from the host chromosome enhances pyocyanin production in all strains tested except for strain CPA0053, which produces low levels of pyocyanin compared with all other strains tested. It is possible that the loss of PfsE in the ∆Pf strains in this study is responsible for the observed increase in pyocyanin production in most strains. As pyocyanin is the terminal signaling molecule in *P. aeruginosa* quorum-sensing networks (28), these results suggest this inhibition is the ultimate goal of Pf phages and may be beneficial to Pf phages during active replication. Pyocyanin and other redox-active phenazines are toxic to bacteria; it is possible that stress responses that are induced by pyocyanin-producing *P. aeruginosa* are detrimental to Pf replication.

We recently discovered that Pf phages encode a protein called PfsE (PA0721), which inhibits PQS signaling by binding to the anthranilate-coenzyme A ligase PqsA, and that this results in enhanced Pf replication (13). One possibility to explain the increased growth rate of LESB58^∆Pf^ could be related to the dramatic increase in pyocyanin production by this strain, which could negatively impact bacterial growth. Further investigation is required to determine if this is true.

Pf lysogens induce avoidance behavior by *C. elegans*, which prefers to associate with the ∆Pf strains. Strikingly, although strains lacking Pf prophages are less virulent in a nematode infection model and attract *C. elegans* in the model described here, the reduced virulence of ∆Pf strains contrasts with their high pyocyanin virulence factor production. This discrepancy may be partly explained by our prior findings that Pf4 suppresses pyocyanin and other bacterial pigment production as a means to avoid detection by innate host immune responses (12) that are regulated by the aryl hydrocarbon receptor (37, 38).

In summary, this research reveals the crucial role of the PflM gene in maintaining Pf lysogeny, demonstrates strain-specific effects on quorum sensing and biofilm formation, reveals the consistent inhibition of pyocyanin production by Pf phages, and suggests a role for Pf phages in protecting *P. aeruginosa* against nematode predation.

## MATERIALS AND METHODS

### Strains, plasmids, primers, and growth conditions

Strains, plasmids, and their sources are listed in Table 2. Unless otherwise indicated, bacteria were grown in lysogeny broth (LB) at 37°C with 230 rpm shaking and supplemented with gentamicin (Sigma) where appropriate, at either 10 or 30 µg mL^–1^.

**TABLE 2:** Strains and plasmids used in this study

Strain or plasmid	Description	Source
*Escherichia coli* strains		
DH5α	Cloning strain	New England Biolabs
S17	Donor strain	(39)
OP50	*C. elegans* food source	PMID 4366476
*P. aeruginosa* strains		
PAO1	Wild type	(14)
PAO1 ∆Pf4	Deletion of Pf4 prophage from PAO1	This study
PAO1 ∆Pf4 ∆Pf6	Deletion of Pf4 and Pf6 prophage from PAO1	This study
LES B58	Liverpool epidemic strain B58	(40)
LES B58 ∆Pf	Deletion of Pf prophage from LESB58	This study
CPA0053	CF clinical isolate	Gift from Paul Bollyky, Stanford University
CPA0053 ∆Pf	Deletion of Pf prophage from CPA0053	This study
CPA0087	CF clinical isolate	Gift from Paul Bollyky, Stanford University
CPA0087 ∆Pf	Deletion of Pf prophage from CPA0087	This study
DDRC3	MDR clinical isolate	Gift from Geetha Kumar, Amrita University
DDRC ∆Pf	Deletion of Pf prophage from DDRC3	This study
*C. elegans* strain		
N2	Wild type	*Caenorhabditis* Genetic Center
Plasmids		
CP59 pBBR1-MCS5 *rsaL-gfp*	GFP *lasI* transcriptional reporter	(41)
CP57 pBBR1-MCS5 *rhlA-gfp*	GFP *rhlA* transcriptional reporter	(41)
CP53 pBBR1-MCS5 *pqsA-gfp*	GFP *pqsA* transcriptional reporter	(42)
CP1 pBBR-MCS5- Empty	GFP empty vector control	(41)
pHERD30T - *pflM*	Cloning vector with *pflM* insert, GmR	This study
pENTRpEX18-Gm:∆*PA0718*	Allelic exchange vector for the deletion of *pflM*	(43)
pLM61	pENTR221L1L2-RFqPCRstandard	This study
pUC57-*rplU*	qPCR standard for *rplU*	(44)

### Construction of deletion mutants

We used allelic exchange to delete alleles from *P. aeruginosa* (43). Briefly, to delete *pflM* (*PA0718),* upstream and downstream homologous sequences (~500 bp) were amplified through PCR from PAO1 genomic DNA using the UP and DOWN primers listed in Table 3. These amplicons were then ligated through splicing-by-overlap extension (SOE)-PCR to construct a contiguous deletion allele. This amplicon was then run on a 0.5% agarose gel, gel extracted (*New England Biolabs #T3010L*), and cloned (Gateway, Invitrogen) into a pENTRpEX18-Gm backbone to produce the deletion construct. The deletion construct was then transformed into DH5α, mini-prepped (*New England Biolabs #T1010L*), and sequenced (Plasmidsaurus.com). Sequencing-confirmed vectors were then transformed into *Escherichia coli* S17 Donor cells for biparental mating with the recipient *P. aeruginosa* strain. Single crossovers were isolated on VBMM (Vogel-Bonner minimal medium) agar supplemented with 30 µg/mL gentamicin, followed by the selection of double crossovers on no salt sucrose. The final obtained mutants were confirmed by excision assay (see below), Sanger sequencing of excision assay products, and whole genome sequencing.

**TABLE 3:** Primers used in this study

Name	Tm (°C)	Sequence
Construction of pENTRpEX18-Gm:∆*PA0718*		
∆*pflM* UP*attB1*-Fwd	62.1	ggggacaagtttgtacaaaaaagcaggcttcCTAATGCCACGAATAGTGACGG
∆*pflM* UP-Rev	65.3	TCAGCCCTCCAGTTGGAATGCGTAGGGACTGGCGGCCAT
∆*pflM* DOWN-Fwd	63.2	GCATTCCAACTGGAGGGCTGA
∆*pflM* DOWN*attB2*-Rev	62.1	ggggaccactttgtacaagaaagctgggtaAAAGTGATTTGTCGGGCGATCC
∆*pflM* Seq-Fwd	57.5	TTTTTGGGGCCGATTTTCTTG
∆*pflM* Seq-Rev	56.3	ATTGGACCGAGGCGTGA
Quantitative PCR		
RF-Fwd	60.5	TAGGCATTTCAGGGGCTTGG
RF-Rev	62.5	GAGCTACGGAGTAAGACGCC
*rplU*-Fwd	52.4	CAAGGTCCGCATCATCAAGTT
*rplU*-Rev	52.6	GGCCCTGACGCTTCATGT
Prophage mutant screening		
16SRNA-F	59.5	TGGTTCAGCAAGTTGGATGTG
16SRNA-R	59.5	GTTTGCTCCCCACGCTTTC
pfsE-F	56.3	ATGCTCCGCTATCTCTCG
pfsE-R	58.4	TCAAACAGCCAGGGAGGC
Excision assays		
Pf4-Fwd1	62.5	GGATATGGAGCGTGGTGGAG
Pf4-Fwd2	59.9	AGTGGCGGTTATCGGATGAC
Pf4-Rev	61.4	TCATTGGGAGGCGCTTTCAT
Pf6-Fwd1	60.5	GTGATCCACGTGTCCAACAG
Pf6-Fwd2	60.5	CCCAGTGCAGATGACTTGGT
Pf6-Rev	60.5	CGCCACTGGTCATTGATCCT
LES B58-Fwd1	59.8	AGCGACAGCCGCCAGCA
LES B58-Fwd2	61.6	GCTTGCCGAACTGCTGGTG
LES B58-Rev	62.5	CGGGTTTCGTCGGTCATCAC
CPA0053-Fwd1	52.8	GCAGGTCGAGGTAGTAG
CPA0053-Fwd2	60	TTCGTCGCTGAACATGACCA
CPA0053-Rev	51.6	CCTCGATCATGTTGAAGT
CPA0087-Fwd1	52.8	GCAGGTCGAGGTAGTAG
CPA0087-Fwd2	60	TTCGTCGCTGAACATGACCA
CPA0087-Rev	51.6	CCTCGATCATGTTGAAGT
DDRC3*gly*-Fwd1	60.1	GCTTTCTACTCCTGAGCATGTA
DDRC3*gly*-Fwd2	59.8	CGCTGCGGAACACCGTG
DDRC3*gly*-Rev	59.5	ACCGTGAAGTACCTGCAGC

### Excision assays

Excision assays were designed as described previously (45). Briefly, a multiplex PCR assay was designed to produce amplicons of distinct sizes if the Pf prophage was integrated (primers Fwd_1 and Rev produce a smaller band) or excised (primers Fwd_2 and Rev produce a larger band) using Phusion Plus PCR Mastermix (Thermo Scientific # F631L). Primers were used at a final concentration of 0.5 µM and are listed in (Table 3).

### Plaque assays

Plaque assays were performed using ΔPf4 as the indicator strain grown on LB plates. Phage in filtered supernatants was serially diluted 10× in phosphate buffered saline (PBS) and spotted onto lawns of PAO1^ΔPf4^. Plaques were imaged after 18 hours of growth at 37°C. PFUs/mL were then calculated.

### Quantitative PCR

Cultures were grown overnight in LB broth with shaking at 37°C. Following 18 hours of incubation, cultures were pelleted at 16,000 × *g* for 5 minutes, washed 3× in 1× PBS, and treated with DNase at a final concentration of 0.1 mg/mL. qPCR was performed using SsoAdvanced Universal SYBR Green Supermix (BioRad #1725270) on the BioRad CFX Duet. For the standard curves, the sequence targeted by the primers was inserted into vectors pLM61 and pUC57-*rplU,* respectively*,* and 10-fold serial dilutions of the standard were used in the qPCR reactions with the appropriate primers (Table 3). Normalization to chromosomal copy number was performed as previously described (44) using 50S ribosomal protein gene *rpIU*.

### Pyocyanin extraction and measurement

Pyocyanin was measured as previously described (20, 46). Briefly, 18-hour cultures were treated with chloroform at 50% vol/vol. Samples were vortexed vigorously, and the organic phase was separated by centrifuging samples at 6,000 × *g* for 5 minutes. The chloroform layer was removed to a fresh tube, and 20% of the volume of 0.1 N HCl was added; then, the mixture vortexed vigorously. Once separated, the aqueous fraction was aliquoted to a 96-well plate, and the absorbance was measured at 520 nm. The concentration of pyocyanin, expressed as μg/mL, was obtained by multiplying the OD_520_ nm by 17.072, as described previously (46).

### Quorum-sensing reporters

Competent *P. aeruginosa* cells were prepared by washing overnight cultures in 300 mM sucrose, followed by transformation by electroporation (47) with the plasmids CP1 PBBR-MCS5 *Empty*, CP53 PBBR1-MCS5 *pqsA-gfp*, CP57 PBBR1-MCS5 *rhlA-gfp*, and CP59 PBBR1-MCS5 *rsaL-gfp* listed in Table 2. Transformants were selected by plating on the appropriate antibiotic selection media. The indicated strains were grown in buffered LB containing 50 mM MOPS (3-morpholinopropane-1-sulfonic acid) buffer and 100 µg mL^–1^ gentamicin for 18 hours. Cultures were then sub-cultured 1:100 into fresh LB MOPS buffer and grown to an OD_600_ of 0.3. To measure reporter fluorescence, each strain was added to a 96-well plate containing 200 µL LB MOPS with a final bacterial density of OD_600_ 0.1 and incubated at 37°C in a CLARIOstar BMG LABTECH plate reader. Prior to each measurement, plates were shaken at 230 rpm for a duration of 2 minutes. A measurement was taken every 15 minutes for both growth (OD_600_) and fluorescence (excitation at 485–15 nm and emission at 535–15 nm). End-point measurements at 18 hours were normalized to cell density.

### *C. elegans* growth conditions

Synchronized adult N2 *C. elegans* were propagated on normal nematode growth medium (NNGM) agar plates with *E. coli* OP50 as a food source.

### *C. elegans* avoidance assays

*C. elegans* avoidance assays were performed as previously described (33). Briefly, synchronized adult N2 worms were propagated at 24°C on 3.5 cm NNGM agar plates with *E. coli* OP50 for 48 hours, collected, and washed 4× to remove residual OP50. NNGM agar was spotted with 20 µL of *P. aeruginosa* (Pf lysogens and their isogenic ∆Pf mutant) overnight cultures (LB broth) as shown in Fig. 7A and grown for 18 hours at 37°C. Worms were plated in triplicate and incubated at 24°C. *C. elegans* migration was monitored hourly for 8 hours.

### Whole genome sequencing and annotation

DNA extraction was performed using NEB Monarch gDNA isolation kits (NEB #T3010). Whole genome sequencing was performed by Plasmidsaurus using an Oxford Nanopore GridION device and corresponding flow cells to >30× coverage. Reads were filtered using Filtlong (v0.2.1) and assembled using Flye (v2.9.1) and/or Velvet (v7.0.4). Contigs were polished using Medaka (v1.8.0) and annotated using Bakta (v1.6.1), Bandage (v0.8.1), and RAST (https://rast.nmpdr.org/). Domain analysis was performed using PfamScan (https://www.ebi.ac.uk/Tools/pfa/pfamscan/) against the library of Pfam HMM using an e-value cutoff of 0.01. Supporting domain models were obtained from the Conserved Domain Database and Defense Finder (48).

### Statistical analyses

Differences between data sets were evaluated with a Student’s *t*-test (unpaired, two-tailed) or two-way ANOVA using the Šidák correction (95% confidence interval threshold) where appropriate. *P* values of < 0.05 were considered statistically significant. GraphPad Prism version 9.4.1 (GraphPad Software, San Diego, CA) was used for all analyses.

## Data Availability

Raw sequencing reads and assemblies for parental strains introduced in this study (Table 2) have been deposited as part of BioProject PRJNA1031220 in the NCBI Sequence Read Archive database.

## SUPPLEMENTAL MATERIAL

Supplemental figures jb.00402-23-s0001.pdf Figures S1 to S8.

ASM does not own the copyrights to Supplemental Material that may be linked to, or accessed through, an article. The authors have granted ASM a non-exclusive, world-wide license to publish the Supplemental Material files. Please contact the corresponding author directly for reuse.
