# Supplementary material for: Targeted deletion of Pf prophages from diverse Pseudomonas aeruginosa isolates has differential impacts on quorum sensing and virulence traits
Source: J Bacteriol. 2024 Apr 30;206(5):e00402-23. doi: 10.1128/jb.00402-23 (PMC11112994; doi:10.1128/jb.00402-23)
Supplement: Supplemental figures — Figures S1 to S8. [file jb.00402-23-s0001.pdf]

Targeted deletion of Pf prophages from diverse *Pseudomonas aeruginosa* isolates has differential impacts on quorum sensing and virulence traits

Amelia K. Schmidt[1], Caleb M. Schwartzkopf[1], Julie D. Pourtois[2], Elizabeth Burgener[2], Dominick R. Faith[1], Alex Joyce[1], Tyrza Lamma[1], Geetha Kumar[3], Paul L. Bollyky[2], and Patrick R. Secor[1#]

[1] Division of Biological Sciences, University of Montana, Missoula, Montana, USA
[2] Division of Infectious Diseases and Geographic Medicine, Department of Medicine, Stanford University School of Medicine, Stanford, CA, USA.
[3] School of Biotechnology, Amrita Vishwa Vidyapeetham, Amritapuri, Kerala, India
[#] Correspondence: Patrick.secor@mso.umt.edu

Supplemental material

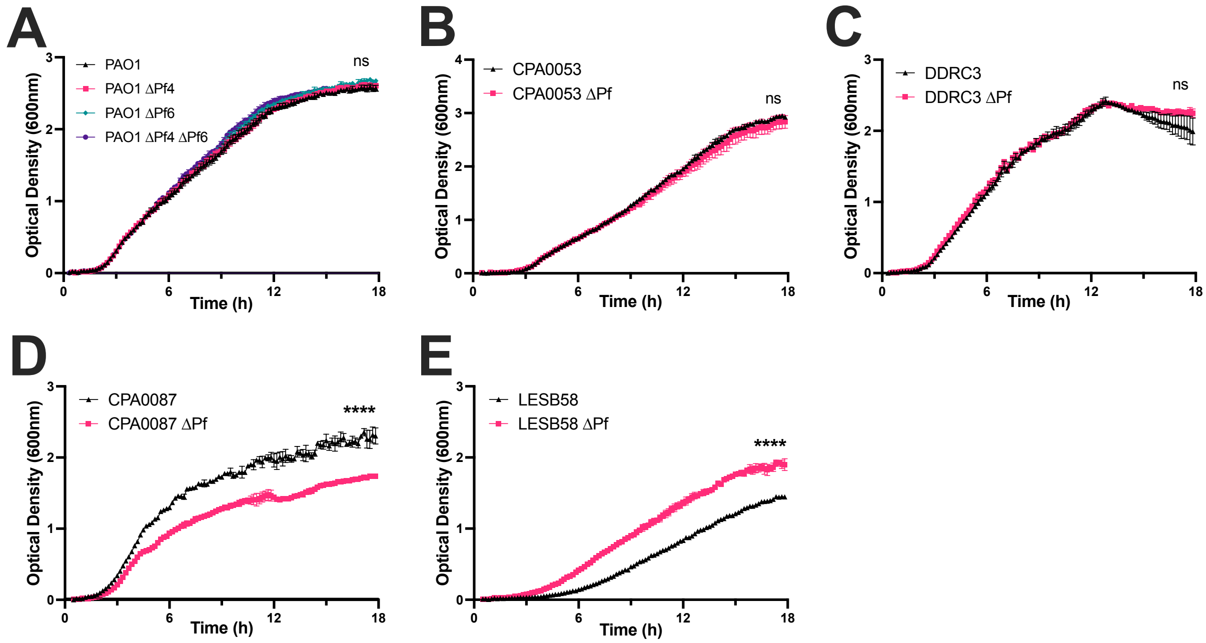

Figure S1. Pf prophages have variable impacts on *P. aeruginosa* growth. Individual colonies were picked and used to inoculate overnight cultures. The following day, overnight cultures were subcultured into fresh LB. Optical density was monitored for 18h, N = 4. Statistical analyses were performed in GraphPad Prism (v10.2.0) by two-way ANOVA, ns, not significant, ****P<0.0001.

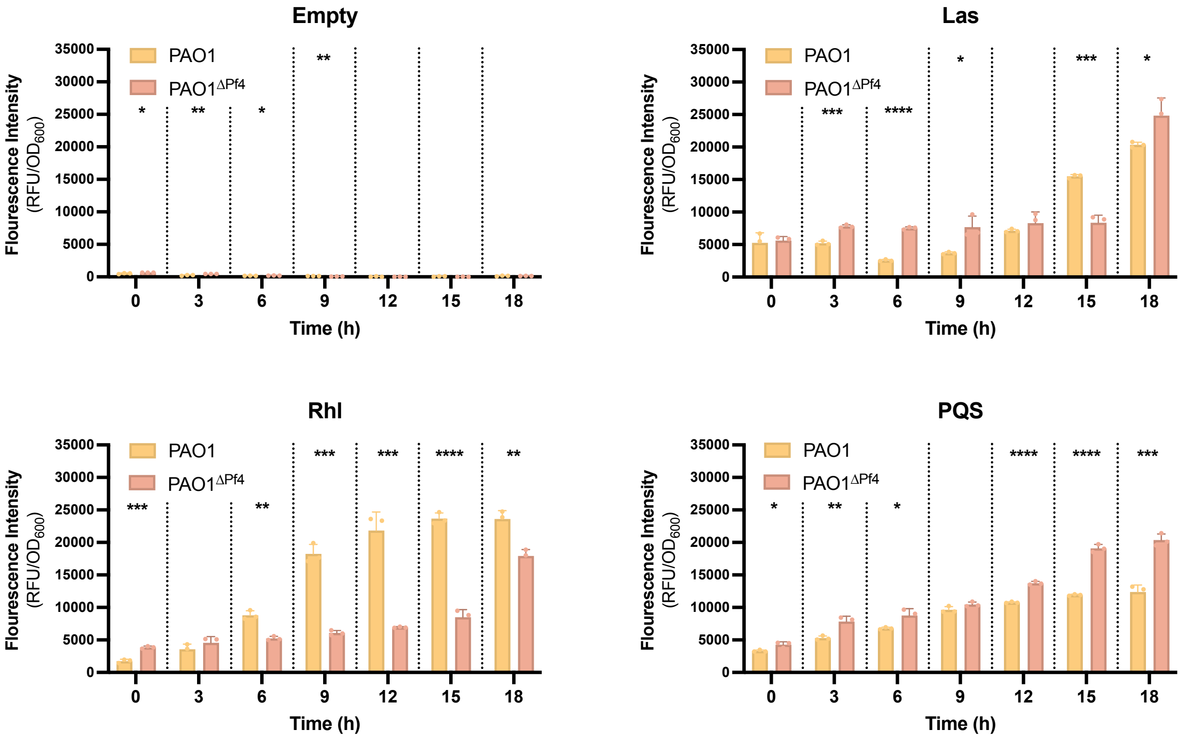

Figure S2: Pf4 modulates *P. aeruginosa* PAO1 quorum sensing. (A-D) Fluorescence intensity from the transcriptional reporters P_{rsaL}-*gfp* (Las), P_{rhlA}-*gfp* (Rhl), or P_{pqsA}-*gfp* (PQS) was measured in the indicated strains after 18 hours of growth. Fluorescence intensity was normalized to cell density (OD_{600}) at each timepoint. Data are the mean ±SEM of three biological replicates. *P<0.05, **P<0.01, ***P<0.001, ****P<0.0001, Student's *t*-test comparing ΔPf strains to the wild-type parent at each time point.

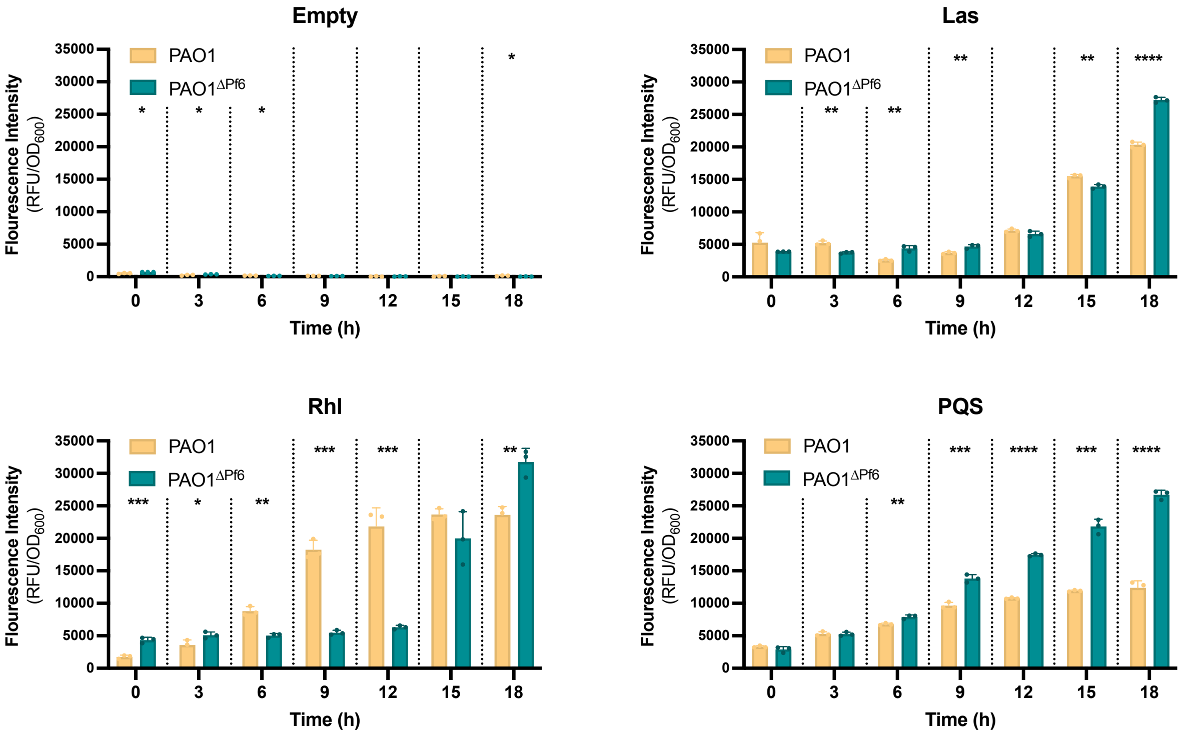

Figure S3: Pf6 modulates *P. aeruginosa* PAO1 quorum sensing. (A-D) Fluorescence intensity from the transcriptional reporters P_{rsaL}-*gfp* (Las), P_{rhlA}-*gfp* (Rhl), or P_{pqsA}-*gfp* (PQS) was measured in the indicated strains after 18 hours of growth. Fluorescence intensity was normalized to cell density (OD_{600}) at each timepoint. Data are the mean ±SEM of three biological replicates. *P<0.05, **P<0.01, ***P<0.001, ****P<0.0001, Student's *t*-test comparing ΔPf strains to the wild-type parent at each time point.

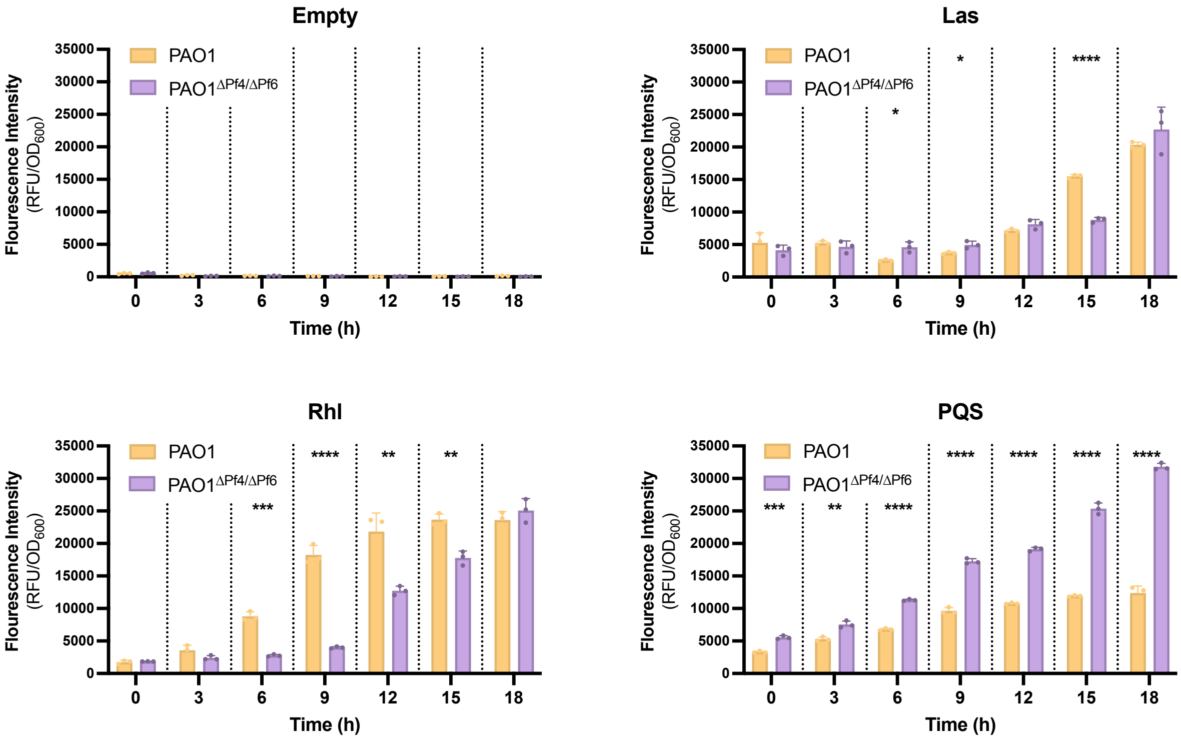

Figure S4: Pf4 and Pf6 modulate *P. aeruginosa* PAO1 quorum sensing. (A-D) Fluorescence intensity from the transcriptional reporters P_{rsaL}-*gfp* (Las), P_{rhlA}-*gfp* (Rhl), or P_{pqsA}-*gfp* (PQS) was measured in the indicated strains after 18 hours of growth. Fluorescence intensity was normalized to cell density (OD_{600}) at each timepoint. Data are the mean ±SEM of three biological replicates. *P<0.05, **P<0.01, ***P<0.001, ****P<0.0001, Student's *t*-test comparing ΔPf strains to the wild-type parent at each time point.

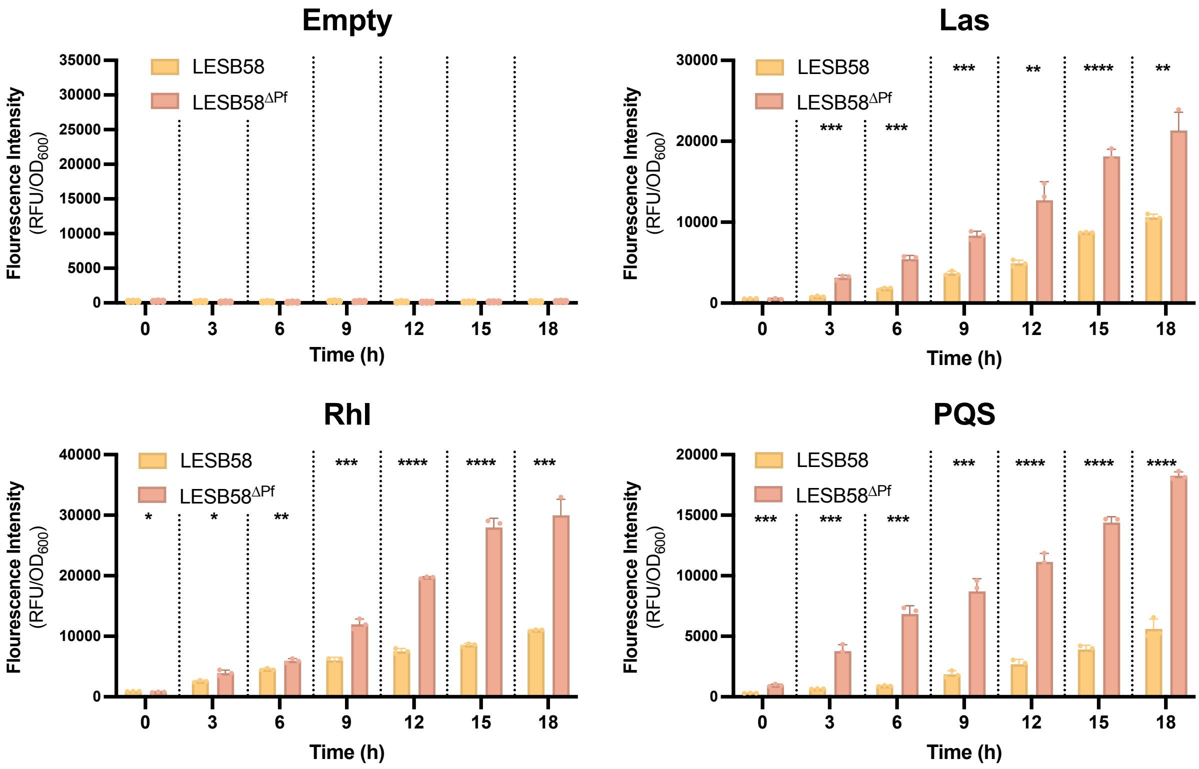

Figure S5: Pf phage modulate *P. aeruginosa* LESB58 quorum sensing. (A-D) Fluorescence intensity from the transcriptional reporters P_{rsaL}-*gfp* (Las), P_{rhlA}-*gfp* (Rhl), or P_{pqsA}-*gfp* (PQS) was measured in the indicated strains after 18 hours of growth. Fluorescence intensity was normalized to cell density (OD_{600}) at each timepoint. Data are the mean ±SEM of three biological replicates. *$P<0.05$, **$P<0.01$, ***$P<0.001$, ****$P<0.0001$, Student's *t*-test comparing ΔPf strains to the wild-type parent at each time point.

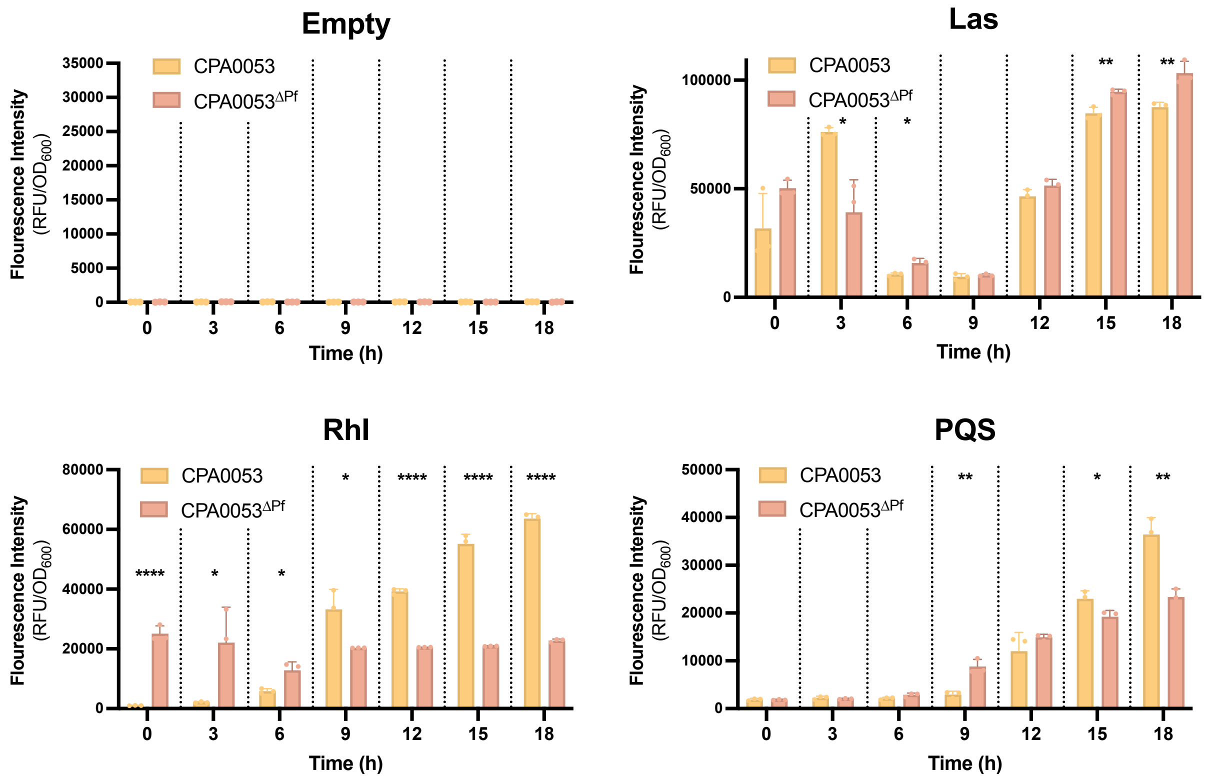

Figure S6: Pf phage modulate *P. aeruginosa* CPA0053 quorum sensing. (A-D) Fluorescence intensity from the transcriptional reporters P_{rsaL}-*gfp* (Las), P_{rhlA}-*gfp* (Rhl), or P_{pqsA}-*gfp* (PQS) was measured in the indicated strains after 18 hours of growth. Fluorescence intensity was normalized to cell density (OD_{600}) at each timepoint. Data are the mean ±SEM of three biological replicates. *P<0.05, **P<0.01, ***P<0.001, ****P<0.0001, Student's *t*-test comparing ΔPf strains to the wild-type parent at each time point.

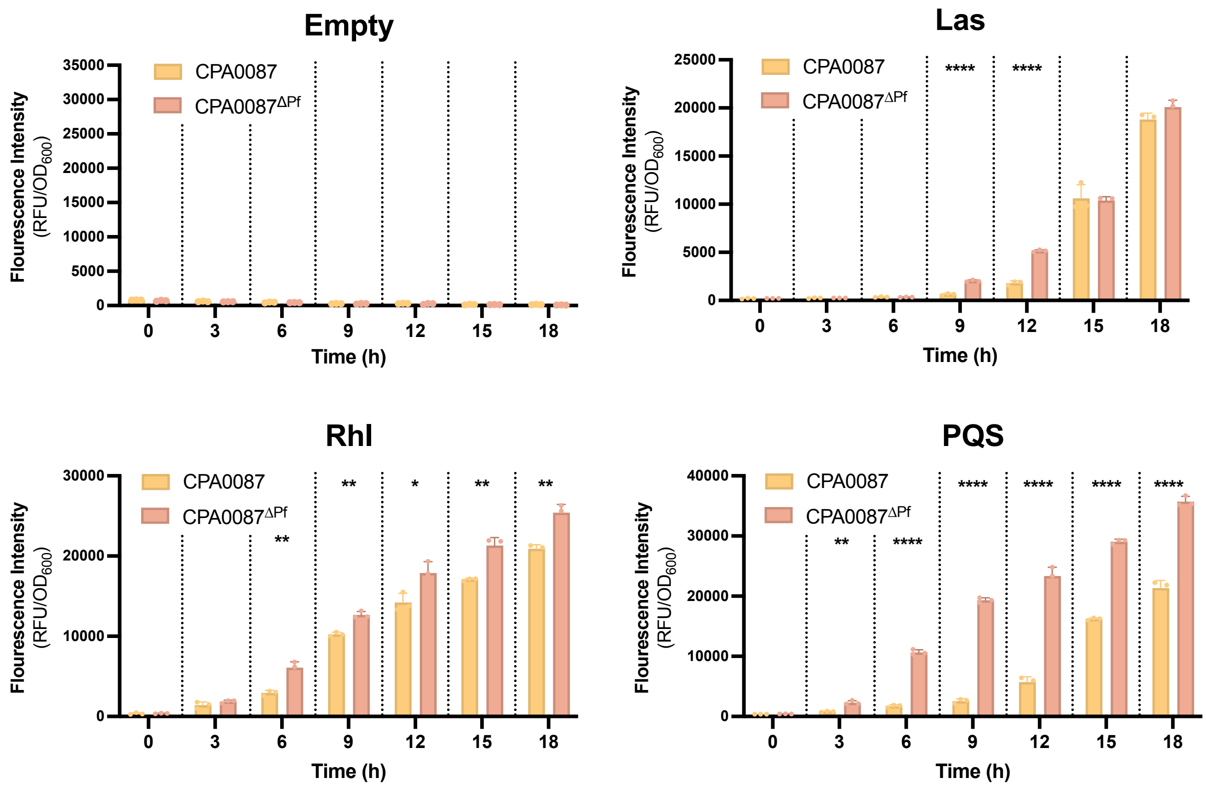

Figure S7: Pf phage modulate *P. aeruginosa* CPA0087 quorum sensing. (A-D) Fluorescence intensity from the transcriptional reporters P_{rsaL}-*gfp* (Las), P_{rhlA}-*gfp* (Rhl), or P_{pqsA}-*gfp* (PQS) was measured in the indicated strains after 18 hours of growth. Fluorescence intensity was normalized to cell density (OD_{600}) at each timepoint. Data are the mean ±SEM of three biological replicates. *P<0.05, **P<0.01, ***P<0.001, ****P<0.0001, Student's *t*-test comparing ΔPf strains to the wild-type parent at each time point.

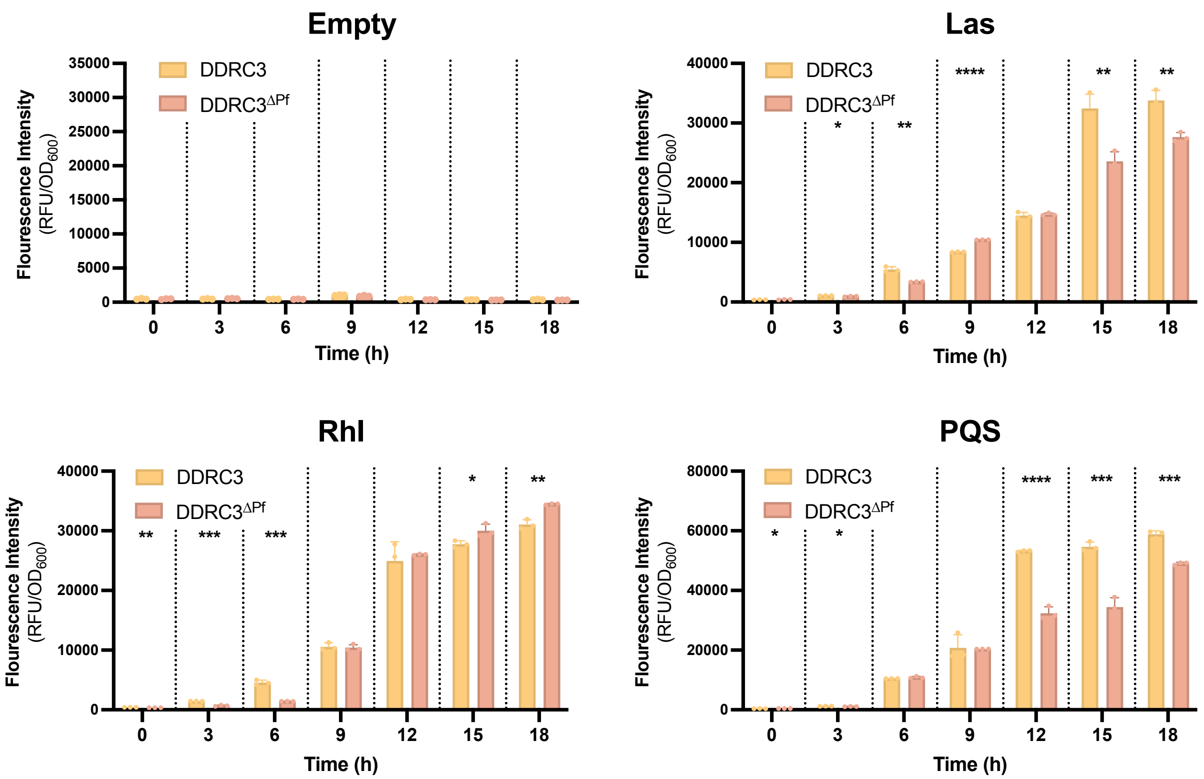

Figure S8: Pf phage modulate *P. aeruginosa* DDRC3 quorum sensing. (A-D) Fluorescence intensity from the transcriptional reporters P_{rsaL}-*gfp* (Las), P_{rhlA}-*gfp* (Rhl), or P_{pqsA}-*gfp* (PQS) was measured in the indicated strains after 18 hours of growth. Fluorescence intensity was normalized to cell density (OD_{600}) at each timepoint. Data are the mean ±SEM of three biological replicates. *P<0.05, **P<0.01, ***P<0.001, ****P<0.0001, Student's *t*-test comparing ΔPf strains to the wild-type parent at each time point.
